# A Mass Causing Right Ventricular Outflow Obstruction - A Dreadful Complication

**DOI:** 10.21470/1678-9741-2018-0314

**Published:** 2019

**Authors:** Rupesh Kumar, Javid Raja

**Affiliations:** 1 Department of Cardiothoracic and Vascular Surgery, Advanced Cardiac Center, Postgraduate Institute of Medical Education and Research, Chandigarh, India.; 2 Department of Cardiothoracic and Vascular Surgery, Advanced Cardiac Center, Postgraduate Institute of Medical Education and Research, Chandigarh, India.

**Keywords:** Heart Neoplasms - Surgery, Rhabdomyoma, Ventricular Septum, Echocardiography, Hemodynamics

## Abstract

The most common cardiac tumour in the pediatric age group is rhabdomyoma. These are usually located in the ventricles, either in the ventricular septum or free wall. Cardiac tumours in early infancy may lead to severely compromised blood flow due to inflow or outflow tract obstruction. The diagnosis of cardiac rhabdomyoma can be established by transthoracic echocardiography (TTE). Rhabdomyomas have a natural history of spontaneous regression; surgical intervention is reserved for patients with symptoms of severe obstruction or hemodynamic instability. In this study, a case of two-year old child who presented with failure to thrive and underwent excision of pedunculated mass from the right ventricular outflow tract was reported.

**Table:** 

Abbreviations, acronyms & symbols	
CT	= Cardiac tumors
MRI	= Magnetic resonance imaging
TTE	= Transthoracic echocardiography

## INTRODUCTION

Rhabdomyomas are by far the most frequent primary tumors of heart in the pediatric age group. They are usually multiple and located within the ventricles but may occur anywhere in the heart. Cardiac tumours may lead to constellation of symptoms in early infancy due to obstruction of blood flow through the inflow or outflow tracts of the heart. Rhabdomyomas have a natural history of spontaneous regression; surgical intervention is reserved for patients with hemodynamic instability. 

## CASE REPORT

A two-year-old baby presented to our emergency department with hurried respiration and failure to thrive for the last one week. Detailed history revealed a hurried breathing, restricted mobility of the baby and reduced appetite over the last one month. The baby was intubated for respiratory fatigue. He was emaciated when examined, with low body weight as compared to babies of his age, afebrile, heart rate of 138/minute, raised jugular venous pressure and mild hepatomegaly. Cardiovascular examinations were essentially normal except a systolic murmur of tricuspid regurgitation. TTE revealed a pedunculated mass approximately 15mmx10 mm attached to the wall of right ventricular outflow tract leading to obstruction of blood flow across it during systole. The pulmonary valve was competent. There was a peak systolic gradient of 70mm Hg across the right ventricular outflow tract. 

**Fig. 1:**
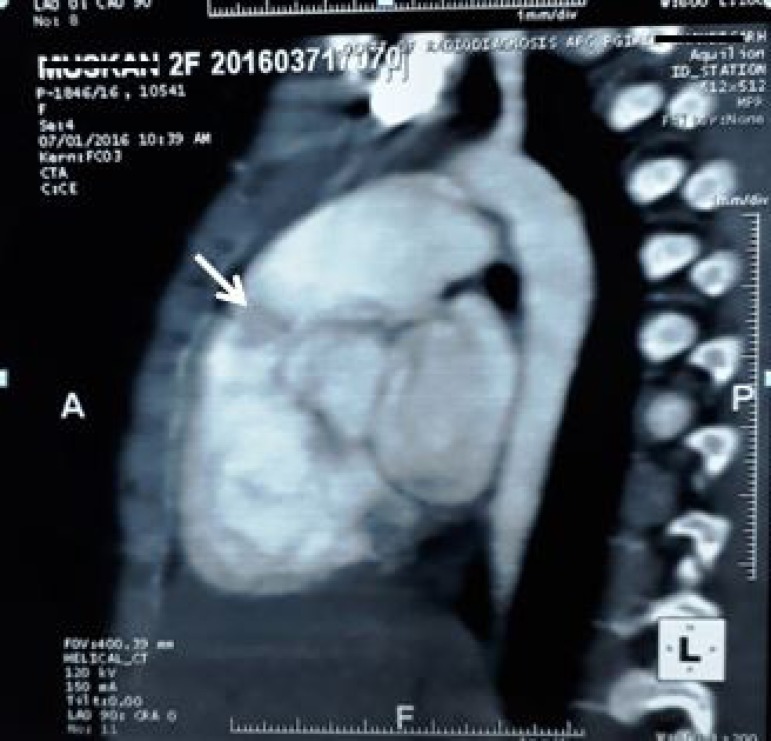
Computer tomography showing right ventricular outflow tract mass.

**Fig. 2:**
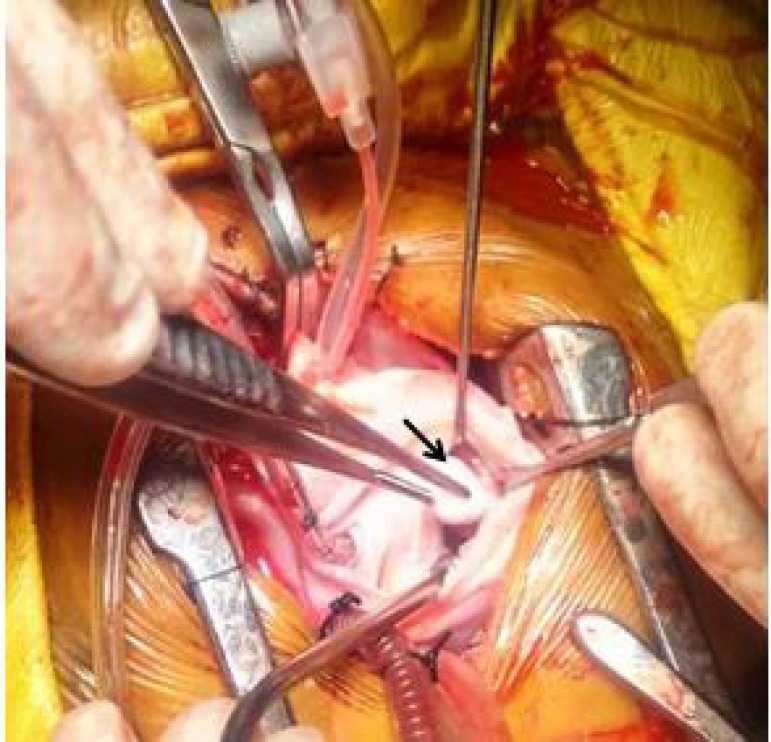
Intraoperative findings showing a pedunculated mass from right ventricular outflow tract.

**Fig. 3:**
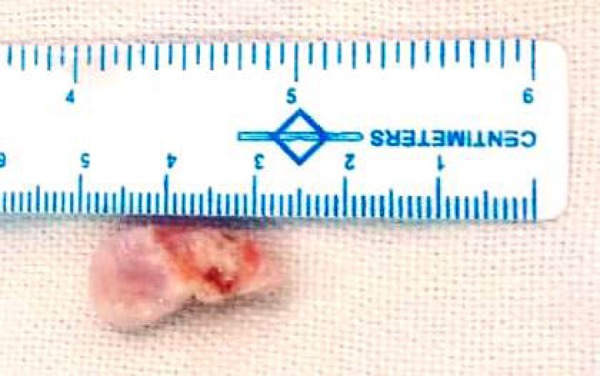
Specimen of pedunculated mass.

Cardiac computer tomogram revealed a pedunculated right ventricular outflow tract mass (Figure 1). The baby was taken for emergency surgery. Through median sternotomy and systemic heparinisation, the baby was cannulated for cardiopulmonary bypass employing direct bi-caval venous cannulation. After cardioplegic arrest a longitudinal right atriotomy was performed, traction sutures were taken anterior to the anterior leaflet of tricuspid valve to allow exposure of right ventricular outflow tract. A pedunculated mass approximately 2cm x 2cm attached to the free wall of right ventricle (Figure 2) about 2 cm proximal to the pulmonary annulus was found and excised with its stalks (Figure 3). It was sent for histopathological analysis.

The cardiopulmonary bypass was reversed and the rest of the procedures were conducted in a usual manner. The patient recovered well and was extubated on the same day. The postoperative TTE showed mild tricuspid regurgitation with no residual mass in right ventricular outflow tract and peak systolic gradients of 10 mm of Hg across it.

The histopathology report came out to be rhabdomyoma. The patient is on follow up for the last two years with serial TTE and there is no recurrence till now. The baby is asymptomatic and gaining weight.

## DISCUSSION

Cardiac rhabdomyoma is the most common primary cardiac tumor accounting for over 60% of all primary cardiac tumors^[1,2]^. Clinical manifestations of cardiac tumours are often non specific and the presenting features of cardiac tumors depend on the size and location of the mass. Cardiac tumours in early infancy may affect the integrity and function of the adjacent cardiac structures leading to severely compromised blood flow due to inflow or outflow tract obstruction, cyanosis, murmur, respiratory distress, myocardial dysfunction, valvular insufficiency, congestive heart failure, arrhythmias, and sudden death^[3,4]^. It can be easily diagnosed on TTE, cardiac CT or MRI. TTE allows accurate location and extent of the mass, the usual feature of such mass is a homogeneous well-circumscribed echo-bright intramural or intracavitary masses that can be found virtually anywhere in the heart but most commonly in the ventricles^[5]^. It is widely accepted that rhabdomyomas have a strong association with tuberous sclerosis and hence brain imaging may also be considered in symptomatic patients^[6]^. Rhabdomyomas usually regress spontaneously and more than 80% of the tumours show complete resolution during early childhood^[6]^. Surgical intervention should be reserved for patients with symptoms of severe obstruction or hemodynamically significant and intractable arrhythmias that are unresponsive to antiarrhythmic drugs^[7]^. The outcome of surgical resection in symptomatic, benign cardiac tumours is favourable^[8]^.

## COMMENT

Rhabdomyomas are the most common primary tumor of the heart in the pediatric age group. They are usually located within the ventricles. Cardiac tumours in early infancy may lead to severely compromised blood flow due to inflow or outflow tract obstruction. The diagnosis of cardiac rhabdomyoma can be established by TTE. Rhabdomyomas may regress on its own; surgery is recommended for severely symptomatic patients. Any pediatric patient who presents with failure to thrive should have a detailed clinical examination and TTE to rule out this surgical emergency. A mass causing obstruction to the right ventricular outflow tract is an extremely rare but curable disease. A prompt diagnosis and surgical management may save the life of such individuals.

**Table:** 

Authors' roles & responsibilities	
RK	Substantial contributions to the conception or design of the work; or the acquisition, analysis, or interpretation of data for the work; drafting the work or revising it critically for important intellectual content; final approval of the version to be published
JR	Substantial contributions to the conception or design of the work; or the acquisition, analysis, or interpretation of data for the work; final approval of the version to be published
